# Antibacterial Properties of Grape Seed Extract-Enriched Cellulose Hydrogels for Potential Dental Application: In Vitro Assay, Cytocompatibility, and Biocompatibility

**DOI:** 10.3390/gels10090606

**Published:** 2024-09-23

**Authors:** Karla Lizette Tovar-Carrillo, Lizett Trujillo-Morales, Juan Carlos Cuevas-González, Judith Virginia Ríos-Arana, León Francisco Espinosa-Cristobal, Erasto Armando Zaragoza-Contreras

**Affiliations:** 1 Instituto de Ciencias Biomédicas, Universidad Autónoma de Cd. Juárez, Av. Benjamín Franklin # 4960, Zona Pronaf, Ciudad Juárez 32315, Chihuahua, Mexico; ltrujillom23@hotmail.com (L.T.-M.); cuevas_gonzalez@hotmail.com (J.C.C.-G.); jrios@uacj.mx (J.V.R.-A.); leon.espinosa@uacj.mx (L.F.E.-C.); 2 Centro de Investigación en Materiales Avanzados, S.C. Miguel de Cervantes No. 180, Complejo Industrial, Chihuahua 31136, Chihuahua, Mexico

**Keywords:** antibacterial, biocompatibility, cytocompatibility, grape seed, hydrogel, mechanical properties

## Abstract

Hydrogels elaborated from *Dasylirion* spp. and enriched with grape seed extract (GSE) were investigated for tentative use in dental treatment. Cellulose-GSE hydrogels were elaborated with varying GSE contents from 10 to 50 wt%. The mechanical and physical properties, antimicrobial effect, biocompatibility, and in vitro cytotoxicity were studied. In all the cases, the presence of GSE affects the hydrogel’s mechanical properties. The elongation decreased from 12.67 mm for the hydrogel without GSE to 6.33 mm for the hydrogel with the highest GSE content. The tensile strength decrease was from 52.33 N/mm^2^ (for the samples without GSE) and went to 40 N/mm^2^ for the highest GSE content. Despite the adverse effects, hydrogels possess suitable properties for manipulation. In addition, all hydrogels exhibited excellent biocompatibility and no cytotoxicity, and the antibacterial performance was demonstrated against *S. mutans*, *E. Faecalis*, *S. aureus*, and *P. aureginosa*. Furthermore, the hydrogels with 30 wt% GSE inhibited more than 90% of the bacterial growth.

## 1. Introduction

The use of hydrogels in the biomedical field has been widely studied and found to be advantageous for various applications [1,2]. Hydrogels are colloidal systems formed by a highly hydrophilic polymeric network, which can be derived from natural sources [2,3]. They have been integrated into multiple biological applications such as tissue engineering, contact lenses [3], and other uses [4]. Furthermore, hydrogels have been utilized for the release of active substances [5,6,7] due to their physical and chemical characteristics, including their ability to swell and their permeability to dissolve different solutes.

Natural polymers have been widely considered for the manufacturing of hydrogels [6,7,8]. These polymers are bioderived materials obtained by physical and chemical methods [7,8,9]. In general, an increase in the use of natural polymers has been noted due to their advantages based on their biological properties, even though their mechanical properties may be moderate [10].

Microbial resistance is a significant worldwide clinical problem, and despite the development of new drugs, the number of microorganisms resistant to antibiotics continues to rise [11]. The pharmaceutical industry has recognized the clinical importance of using plants as natural alternatives for developing new drugs to control multi-resistant microorganisms [12,13,14,15]. Some plants are rich in antibacterial agents such as flavonoids, alkaloids, terpenoids, tannins, saponins, and phenols [15,16]. It has been reported that grape seed extract (GSE) possesses antibacterial activity against common oral cavity bacteria such as *S. mutans*, *E. Faecalis*, *S. aureus*, and *P. aureginosa*. GSE contains flavonoids and phenolic compounds that show an inhibitory action against bacterial biofilm formation by affecting the protein transport mechanism into the inner cytoplasmic membrane of the bacteria [17,18]. Once flavonoids and phenolics are found intracellularly, they reversibly bind to the 30s subunit of the bacterial ribosome [19]. This action prevents the binding of tRNA (transfer ribonucleic acid) to the mRNA (messenger ribonucleic acid) in the ribosomal complex, thereby inhibiting protein synthesis in bacteria [18]. Thus, GSE represents an alternative to traditional antibacterial agents like chlorhexidine and possesses wound-healing properties that are not offered by dental bactericides [20]. Consequently, these functions of GSE make it a compelling choice for dental applications.

It is well known that wound healing is a complex process, and if not treated properly, it can have serious consequences. Additionally, the materials currently used can also impact the process. Therefore, finding biocompatible materials with properties that promote and speed up the healing process is essential. Some studies have suggested that hydrogels containing natural extracts have the potential for application in the medical field. Khanna et al. reported the outcome of grape seed extract on in vivo wound healing in rat models [21]. They revealed that GSE accelerates wound healing (*p* < 0.05), improves the histological structure and connective tissue deposition, and increases proliferation-stimulated growth factors. Furthermore, Hemmati et al. found that GSE enhanced the wound contraction and closure process in rabbits, with wound healing occurring within 15 days [22]. They used GSE in cesarean sections and reported that accelerated wound healing reduced the closure time. These studies confirm the wound-healing properties of GSE and its rapid clearance from systemic circulation. 

This work aimed to create a cellulose hydrogel that contains grape seed extract with antibacterial properties. This hydrogel could be used as an alternative to utilize the waste from the production of sotol, a traditional alcoholic beverage from Northern Mexico. The study analyzed how different amounts of grape seed extract in the hydrogels affected antibacterial activity, mechanical properties, cytotoxicity, and biocompatibility. If successful, these grape seed extract-enriched hydrogels could potentially be used as surgical dressings in dentistry.

## 2. Results and Discussion

### 2.1. Hydrogel Preparacion and Characterization

**Figure 1:**
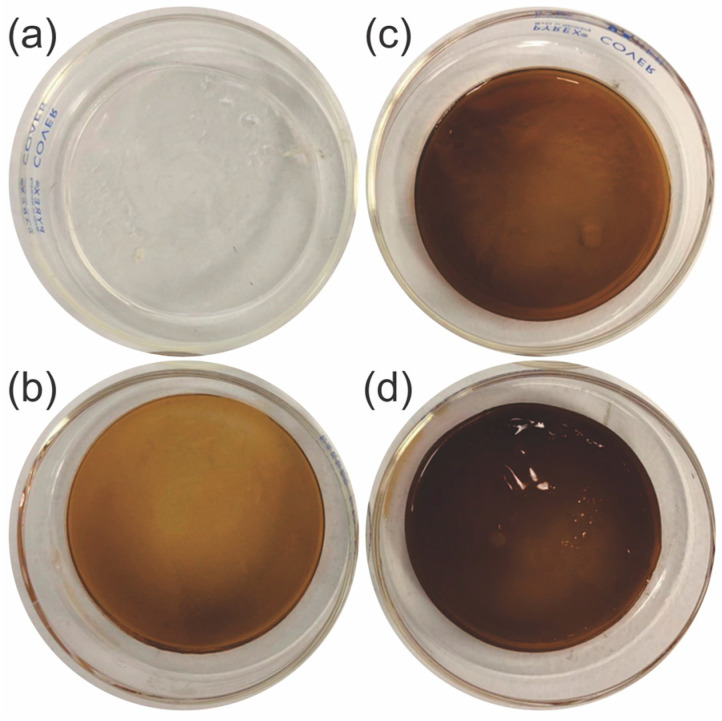
Hydrogel films of (**a**) cellulose control, (**b**) 90/10, (**c**) 70/30, and (**d**) 50/50 (cellulose/GSE) in wet conditions.

Hydrogel films were elaborated from cellulose and GSE solutions. The GSE content ranged from 10 to 50 wt%, and the films were produced in an ethanolic atmosphere using an inverse phase method. Figure 1 shows images of the hydrogels. It was observed that the higher the GSE content, the darker the color of the films. Additionally, as the GSE content increased, the films appeared to be softer than the colorless cellulose hydrogel control.

The results of the mechanical tests and physical properties of the hydrogel films are reported in Table 1. The equilibrium water content (EWC) increased with the increment of GSE. This tendency was also observed in the elongation. In this test, the elongation decreased from 12.67 mm for the film with only cellulose to 6.33 mm for the hydrogel with 50 wt% GSE. Similar behavior was observed with the contact angle measurements. Due to the increment of water content in the hydrogel with the increment of GSE, a significant decay of tensile strength and shear viscosity was observed. For tensile strength, a resistance of 52.33 N/mm^2^ was calculated for the films with 100% cellulose, while for the samples with 50 wt% GSE, a decrease to 40 N/mm^2^ was observed. In addition, a similar tendency was registered for shear viscosity, with a decrement of 410.67 to 363.67 with the increment of GSE in the hydrogel. 

**Figure 2:**
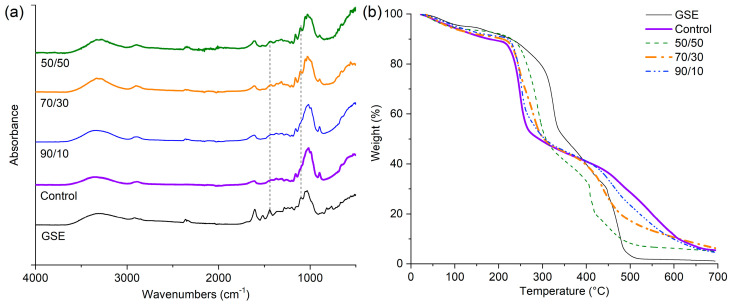
(**a**) FT-IR spectra of GSE’s pure and hydrogel films in dry conditions, and (**b**) thermogravimetric analysis of pure GSE and hydrogel formulations.

The hydrogel films were analyzed using FT-IR for chemical group analysis. Figure 2a shows the FT-IR spectra of pure GSE, cellulose hydrogel, and SGE-enriched hydrogels. The spectrum of GSE exhibits absorption peaks at 3290 cm^−1^, corresponding to the vibrations of the O-H bond from polysaccharides and lignins. In addition, absorptions near 2920 and 2850 cm^−1^ are ascribed to the asymmetric and symmetric vibrations of the C-H bonds of the methylene groups. The peak at 1605 cm^−1^ is related to the carboxyl groups belonging to pectins and phenolic compounds [23]. In addition, the vibrations 1520, 1440, and 1099 cm^−1^ are related to the C-C and C-H bonds of the polysaccharides and phenolic compounds [24]. Finally, the peak at 1033 cm^−1^ is ascribed to the glycosidic structure of cellulose. Spectra of the pure cellulose and enriched hydrogels presented a broad peak centered at 3400 cm^−1^ due to the stretching vibration of the bonded and non-bonded hydroxyl groups. The vibrations of C-O-C and C-O of the polysaccharide structure in the *Dasylirion* spp. hydrogel (control) were found in the region between 1200 cm^−1^ and 900 cm^−1^ [25]. In general, the intensity of the peaks at 1520, 1440, and 1099 cm^−1^ increases as the GSE content augments in the hydrogel, indicating a rise in the composition of phenolic compounds. The described peaks are related to the compounds reported in the GSE composition by HPLC [26,27].

Figure 2b illustrates the TGA traces of the GSE, control, and enriched hydrogels. The GSE presents a continuous weight loss from room temperature to 200 °C (loss of ~10%). This transition was attributed to the evaporation of water and low molecular weight compounds. A second transition was observed between 200 and 330 °C (loss of ~50%), corresponding to the degradation of the polymeric structure of cellulose and lignin. As for the control and GSE-enriched hydrogels, the thermograms show similar patterns to the pure GSE. Two important weight losses were also observed. The first ranges from ambient to 200 °C (loss of ~10%), corresponding to the evaporation of residual water, solvent traces, and cellulose oligomers. The second transition, from 200 to 300 °C (loss of ~50%), is attributed to the thermal decompositión of the cellulose backbone, and the transitions at higher temperatures correspond to the remaining carbon [28]. The GSE traces indicate higher thermal stability (from 200 to 350 °C) regarding the pure cellulose control. Such a difference was related to the lignin contained in the grape seed since this component is thermally degraded in a wider temperature range than cellulose. Furthermore, it was observed that with an increasing GSE content, this transition exhibits a shift to higher temperatures, indicating that the GSE provides a positive effect on the thermal stability of the enriched hydrogel. Similar results were reported for Astrini et al. and Jia et al., who tested cellulose hydrogels [29,30]. The thermal analysis results align with those of FT-IR, showing progressive changes linked to the GSE content. An important aspect of the thermal stability of the enriched hydrogels is that the degradation transitions occurred at temperatures far from the human body temperature. Additionally, the hydrogels did not show reactivity between the phases, so no reactivity with human tissue would be expected. These characteristics make enriched hydrogels promising materials for potential use in dentistry. 

**Figure 3:**
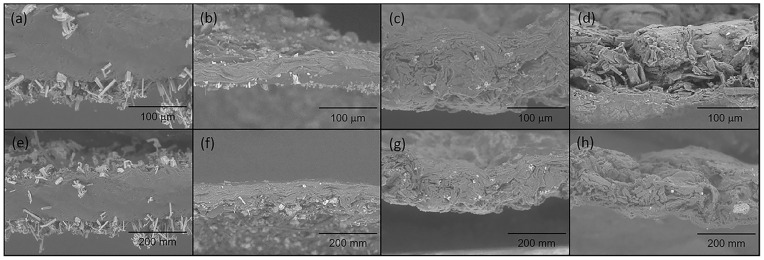
SEM images of a pure cellulose hydrogel (**a**,**e**) and formulated with GSE at 10 wt% (**b**,**f**), 30 wt% (**c**,**g**), and 50 wt% (**d**,**h**).

Figure 3 portrays the SEM images of the hydrogel films without GSE (a,e) and containing 10 (b,f), 30 (c,g), and 50 wt% GSE (d,h). All films show relatively homogenous surfaces, suggesting that the GSE is distributed uniformly into the cellulose matrix—an important aspect of good mechanical performance. Roy et al. and Ji et al. showed similar results when testing cellulose enriched with grape seed and grape seed extract, respectively [31,32].

### 2.2. Antimicrobial Assay

**Figure 4:**
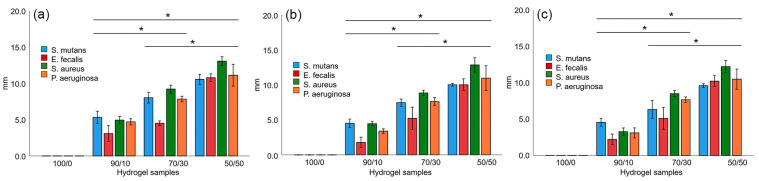
Inhibition halo at (**a**) 15 h, (**b**) 24 h, and (**c**) 48 h culture time at 37 °C. Data presented in millimeters, mean ± standard deviation, and error bars 95% CI. * indicate statistical differences between the concentration groups.

Antimicrobial tests were conducted using several techniques. As for the inhibition halo assays, four bacteria that are associated with the oral cavity were studied. Figure 4 shows the inhibition halo’s size in mm at the following different incubation times: (a) 15 h, (b) 24 h, and (c) 48 h. As noticed, the inhibition halo was present for the cellulose hydrogel without the GSE. On the other hand, the GSE-enriched hydrogels developed an inhibition halo against *S. mutans*, *S. faecalis*, *S. aureus*, and *P. aeruginosa* [33,34,35,36], showing a greater effect as the GSE content increased. This demonstrated that the higher the GSE content, the greater the size of the inhibition halo, which was consistently observed for all hydrogel formulations at all culture times. This was associated with the rich polyphenol content in GSE. It is worth saying that several approaches have reported the antibacterial activity of GSE against some of the bacterial species tested in this study [36,37,38,39].

Table 2 enlists the antibacterial effect measurements by colony-forming units (CFUs) using *S. mutans*, *S. faecalis*, *S. aureus*, and *P. aeruginosa*. The entirety of the microorganisms were sensitive to the various hydrogel formulations containing GSE [31,32,33,34,35,36,37]. However, the hydrogel of pure cellulose showed the highest CFU count, which was similar to the dish inoculated with only bacteria and the dish without hydrogel. This evidences the non-antibacterial activity of the tested hydrogels of cellulose alone. On the other hand, a significant decrease in CFUs was observed in the hydrogels containing 30 and 50 wt% GSE. For instance, with a 30 wt% GSE content, 36 CFUs were found for *S. mutans*, and barely a CFU count was observed for *E. fecalis*, *S. aereus*, and *P. aeruginosa* [36,37,38,39]. More than 90% inhibition of bacterial growth was observed in the samples with 30 wt% GSE. Concerning the hydrogel with 50 wt% GSE, it developed the highest antibacterial effect, decreasing almost to zero with the CFU counts for *S. mutans*, *E. faecalis*, *S. aereus*, and *P. aeruginosa*. Such inhibition of bacterial growth is as high as that observed with chlorhexidine or other dental biocides but has the advantage of fewer adverse reactions in the oral cavity, e.g., inflammation of gums, dry mouth, and others [20]. As found, the antibacterial effect was related to the presence and content of GSE in the hydrogels. As described above, GSE inhibits protein synthesis in bacteria, an effect that is emphasized by increasing the GSE. The antibacterial property, without the possible side effects caused by conventional antibacterial agents, is important for dental applications.

### 2.3. Cytotoxicity and Biocompatibility Assays

#### 2.3.1. Protein Adsorption Assay

**Figure 5:**
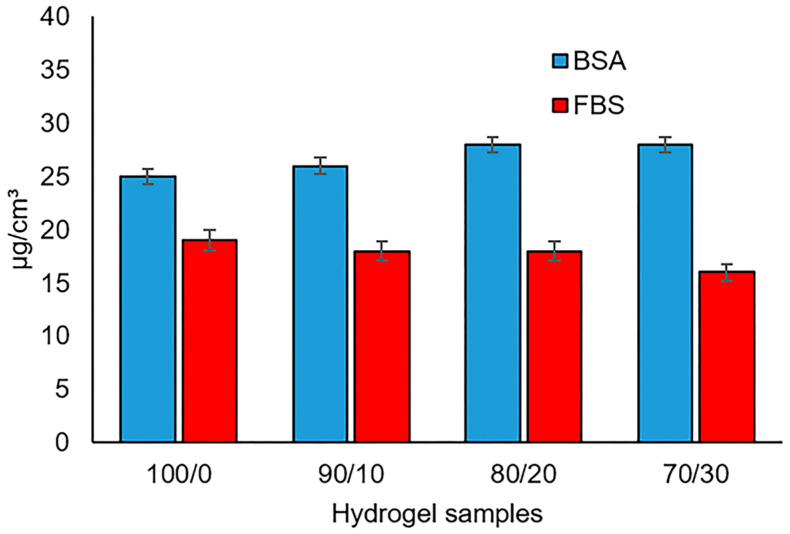
Protein adsorption on hydrogel films (data are presented in mean ± standard deviation), indicating statistical differences between the concentration groups of GSE *p* < 0.05.

For biocompatibility achievement, the GSE-enriched hydrogel serum protein assay was carried out with BSA (bovine serum albumin) and FBS (fetal bovine serum). Figure 5 shows a progressive rise in protein adsorption for BSA as the GSE content increases. This was attributed to the augment in the stiffness of the hydrogel surface with increasing GSE, which promotes more BSA adsorption. On the other hand, the FBS decreased with the increment of GSE; similar results on BSA and FBS adsorption were reported by Tamada et al. [40]. This was associated with the contact angle decrement as a function of the GSE content. It has been reported that BSA prefers hydrophilic surfaces, while FBS shows the opposite tendency [41].

#### 2.3.2. Cell Adherent Number

**Figure 6:**
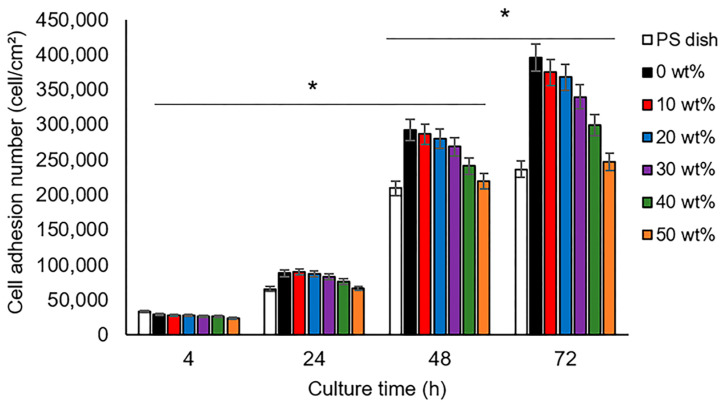
Cell adherent number on PS dish (control) and GSE-enriched hydrogel films with different GSE contents. * indicate statistical differences between the concentration groups of GSE *p* < 0.05 and *p* < 0.01, respectively.

For applying GSE-enriched hydrogels in dentistry, fibroblast adhesion on the hydrogel surface is a major factor. To evaluate such a behavior, microscope images of adherent fibroblasts on the hydrogel film’s surface were used to calculate the cell adherent numbers. Figure 6 indicates a decreasing trend in the adherent cell number with the increment of GSE, compared with the value found on the PS (polystyrene) dish used as the control. In the first 4 h of culture, more adherent cells were observed on the hydrogels without the GSE. Nevertheless, no significant difference in the cell adherent number was found for the GSE-enriched hydrogels. At 24 h of culture, a higher cell adherent number was evidenced in all samples, and after 48 and 72 h, significant differences in the cell adherent number were observed. For 24, 48, and 72 h, a slight decrease trend with the increment of GSE is noted, and the PS dish registered the lowest value. Moreover, the highest cell adherent number was found on all the hydrogels without GSE. The results showed that the surface of the GSE-enriched hydrogels has the necessary characteristics for protein adsorption and cell adhesion. Adherent cells can grow and develop to promote cell proliferation and, subsequently, tissue regeneration—a property applicable in dental treatments.

**Figure 7:**
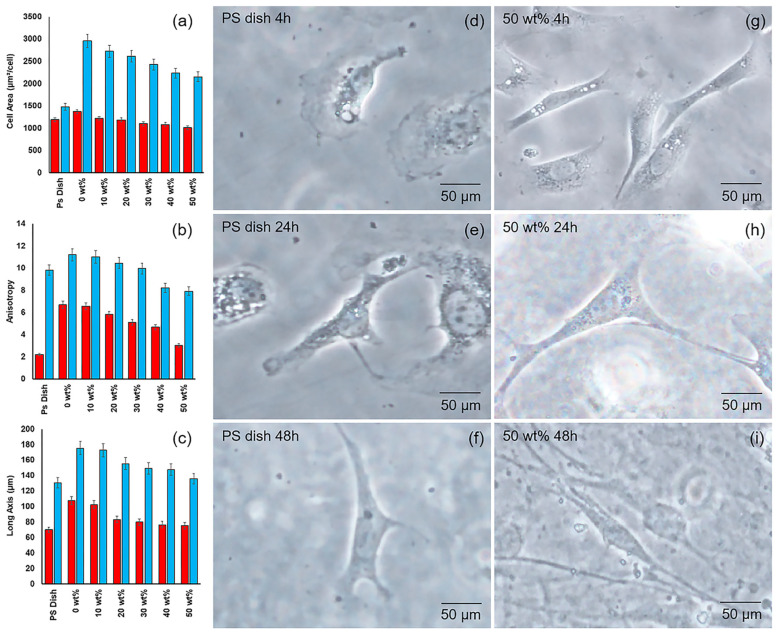
Cytocompatibility of adherent cell on GSE-enriched hydrogels. (**a**) Cell area, (**b**) anisotropy, and (**c**) aspect ratio. Red bars indicate 4 h of cell culture and Blue bars indicate 72 h of cell culture. Phase-contrast light images of adherent cells on GSE-enriched hydrogel films’ varying culture times. Adherent cells on the PS dish (control) at (**d**) 4 h, (**e**) 24 h, and (**f**) 48 h. GSE-enriched hydrogel with 50 wt% at (**g**) 4 h, (**h**) 24 h, and (**i**) 48 h.

Figure 7 shows the cell morphological parameters, such as (a) the cell area, (b) anisotropy, and (c) aspect ratio, of the adherent cells on the GSE-enriched hydrogels. All data were obtained from the cell adherent images using the Cellsens software 4.2 version. The morphology parameters depend on the material surface properties [42,43]. The figure indicates a decrease in morphology parameters with the increment of GSE. As described above, increasing the GSE affected the surface properties of the hydrogel, in turn influencing the cellular morphology of the adherent cells. Figure 7a–c shows the cell area, anisotropy, and aspect ratio with the increment of GSE. Similar results reported that the stiffness and smoothness of surfaces impact the cell morphological aspects of adherent cells [43,44,45]. This was accredited to protein adsorption, depending on the GSE content. In addition, Figure 7d–i shows the phase-contrast images of the adherent cells on the PS dish (control) and GSE-enriched hydrogel’s surface, with the 50 wt% GSE at various culture times. Remarkable differences are noted in the cell morphology of the adherent cells on the PS dish and GSE-enriched hydrogel, especially in the first 4 h of culture. Figure 7d shows semi-circular-shaped cells, compared with the more anisotropic elongated cells seen in Figure 7g. This indicates that the cells adhered more tightly to the surface, showing an early characteristic anisotropic shape, observed and reported for fibroblast cells [46]. Figure 7g–i displays anisotropic elongate-shaped cells compared to Figure 7d–f, which shows a less anisotropic elongated shape at 24 h of cell culture. Similar behavior was reported in our previous work [23]. In addition, similar results were obtained in the hydrogel films despite the negative effect observed on the mechanical properties due to the presence of GSE in the hydrogel in this assay. Other works reported the influence of mechanical properties on cell adhesion and mechanotaxis due to the decrement of film stiffness [40,46]. It is important to mention that an anisotropic shape was observed in all GSE films. This suggests the advantage of GSE-enriched hydrogels, which provide a more suitable surface for cell adhesion than the PS dish since the enriched hydrogel developed a cell morphology similar to mature fibroblasts, even in the first hours of culture. Furthermore, the morphology of the adherent cells showed no notable difference, even with an increasing GSE in the film. This is in agreement with the previous results [20].

## 3. Conclusions

GSE-enriched hydrogels showed antibacterial properties against *S. mutans*, *E. faecalis*, *S. aureus*, and *P. auroginosa,* and the cytocompatibility and biocompatibility were not affected by the addition of GSE. No remarkable differences in the cell adhesion number, cell area, aspect ratio (anisotropy), and long axis were observed on the adherent cells as a function of the GSE content. However, although elongation and the tensile strength decreased, perfectly manipulable hydrogels with antibacterial properties against a series of common bacteria in the oral cavity were obtained. The GSE hydrogels showed a more suitable surface for cell adhesion than the commercial dish for cell culture assays. The cells on the GSE-enriched hydrogel surface showed more anisotropic and adherent bounds, even in the first 4 h of culture. This research allows us to conclude that incorporating grape seed extract into the cellulose hydrogel obtained from *Dasylirion* spp. presents an appropriate antibacterial activity against pathogens commonly found in the oral cavity. In addition, the cell adhesion and mechanical properties make the enriched hydrogels a promising material for conducting a study for treating dental wounds.

## 4. Materials and Methods

### 4.1. Materials

The fibers of *Dasylirion* spp. were obtained from the waste from the manufacturing process of sotol, an alcoholic drink similar to tequila, typical of Chihuahua State, Mexico. The grape seed extract (GSEs, food grade, ≥95.0%. Standardized extract with 95% polyphenols) was bought from Zazzee Naturals (Austin, TX, USA), and N,N-Dimethylacetamide (DMAc), lithium chloride, ethanol, sodium hydroxide, sodium hypochlorite, and sulfuric acid were purchased from Sigma-Aldrich (St. Luis, MO, USA). The bicinchoninic acid (BCA) kit, fetal bovine serum (FBS), and bovine serum albumin (BSA) were delivered from Sigma-Aldrich (St. Luis, MO, USA), and phosphate-buffered saline (PBS, Dullbecco Co., Ltd., St. Luis, MO, USA), 0.05 *w*/*v*% trypsin-0.053 M-ethylenediaminetetraacetate (trypsin-EDTA), formaldehyde (37 vol% aqueous solution), and phosphate-buffered saline (PBS) were purchased from Chemical Tech Co., Ltd. (Mexico City, Mexico). For the cell culture experiments, NIH3T3 mouse embryonic fibroblast cells were delivered from Vitrogen (Tokyo, Japan).

### 4.2. Hydrogel Preparation

To prepare the cellulose solutions, *Dasylirion* spp. fibers were chemically treated according to our previous report [19]. Then, the treated cellulose fibers were exposed to a solvent exchange system with DMAc/LiCl, as reported previously [40]. To obtain the grape seed extract (GSE)-enriched cellulose hydrogels, the GSE was dried for 72 h at 70 °C in an oven, then a GSE 10 wt% solution was obtained using DMAc/LiCl (6 wt% LiCl) in an Erlenmeyer flask and stirred for 3 days to promote GSE dissolution. Then, the solution was decanted to eliminate the non-soluble extract. The cellulose and GSE solutions were mixed to achieve the hydrogel films. The GSE content varied from 10 to 50 wt%, according to the literature [32]. The GSE-enriched hydrogels were obtained by pouring 10 g of cellulose-GSE solution into a glass tray in an ethanolic atmosphere overnight. To remove the solvent residues, the cellulose-GSE films were washed three times with distilled water in a shaking bath for 36 h. The films were then stored in PBS at 4 °C.

### 4.3. Hydrogel Characterization

The mechanical properties of the hydrogel films were evaluated. The tensile strength (σ) and elongation (ε) were measured using a LTS-500N-520 (MinebeaMitsumi, Tokyo, Japan) universal testing machine with a 2.5 kN cel. Film samples (1 mm × 10 mm × 50 mm) were cut and uniaxially deformed along the longest axis. For each hydrogel formulation, five measurements were made and averaged. In total, three experiments for each GSE content were performed. σ and ε were calculated by Equations (1) and (2), respectively.

(1) $$\sigma (\frac{N}{{\mathrm{mm}}^{2}})=\frac{F}{A}$$

(2) $$\varepsilon (\%)=\left(\frac{∆L}{L_{i}}\right)\times \;100$$

where F and A stand for the maximum load (N) and cross-sectional area (mm^2^), respectively, ΔL is the length difference (Final length − Initial length), and L_i_ is the initial length.

As for the films’ water swelling, the equilibrium water content (EWC) was evaluated. The weights of the dried and hydrated samples were registered as follows: hydrogel samples (5 mm × 5 mm) were cut and immersed in distilled water for 36 h. Afterward, the samples were removed and rinsed for excess water, and the weight of the hydrated film was registered as per Equation (3).

(3) $$\mathrm{EWC}(100)=\left[\left(\frac{W_{s}-\;W_{d}}{W_{s}}\right)\right]\times 100$$

W_s_ and W_d_ stand, respectively, for the weight of the swollen samples and the dry weight of the sample.

For the contact angle measurements (DMo-502WA, Kyowa Interface Science Co., Ltd., Saitama, Japan), 3 μL of distilled water was dropped onto the hydrogel samples (20 mm × 20 mm). The angle that formed between the water drop and the film’s surface was measured. For each formulation, three hydrogel samples were analyzed, and the data were averaged.

A Fourier-transform infrared spectrometer (FT-IR) (SA ALPHA, Bruker, Billerica, MA, USA) was used to characterize the functional groups in the hydrogel. The samples were analyzed in the range from 4000 to 400 cm^−1^, with an average of 30 scans. The hydrogels were dried for 72 h before analysis.

Regarding thermal stability, the hydrogels were dehydrated and tested using a thermogravimetric analyzer (SDT Q600, TA Instruments, New Castle, DE, USA) with a sensitivity of 0.1 g. Measurements were carried out with 10 mg of the sample, heating from room temperature to 800 °C under an air atmosphere at a heating rate of 10 °C min^−1^.

The hydrogel’s microstructure was analyzed by a scanning electron microscope (SU3500, Hitachi, Tokyo, Japan) at 15 KV. The tested samples were dried in a freeze dryer (BK-FD10PT, BIOBASE, Jinan, China) at −60 °C and in a vacuum atmosphere for 24 h.

### 4.4. Antimicrobial Assay

For the hydrogel antimicrobial tests, *Streptococcus mutans* ATCC 25923, *Enterococcus faecalis* ATCC 29212, *Pseudomonas aeruginosa* ATCC 15442, and *Staphylococcus aureus* ATCC 29213 were used [19,47]. The bacterial concentration was determined using a spectrophotometer (7305, Jenway, Vernon Hills, IL, USA) to determine the optical density at 600 nm, and the inoculum was standardized to an optical density of 0.1 absorbance units.

Regarding the inhibition halos, Muller–Hinton agar was prepared and sterilized at 120 °C and placed in Petri dishes. Subsequently, circles of hydrogel (5 mm diameter) were cut from each formulation. Cellulose hydrogel film without the GSE was used as a control in each tested group. Therefore, all circles of hydrogel were sterilized under UV light for 25 min. For the antimicrobial assay, 100 µL of inoculum was placed in each culture medium dish and spread over the entire surface using sterile cotton-tipped applicators. Immediately, the hydrogel samples were placed in the inoculated Petri dish and incubated for 24 h at 37 °C. Finally, the inhibition halos were measured at 15, 24, and 48 h of culture time.

Complementarily, growth dynamics was carried out by turbidimetry using tubes with Muller–Hinton broth inoculated with *Streptococcus mutans*, *Enterococcus faecalis*, *Pseudomonas aeruginosa*, and *Staphylococcus aureus*. As a control, tubes with broth and hydrogel without inoculum were used. The bacterial concentration was determined using the UV–vis spectrophotometer at 600 nm (7305, Jenway, Vernon Hills, IL, USA), and the inoculum was standardized to an optical density of 0.1 absorbance units. The hydrogel films were dried at 70 °C and subsequently immersed in ethanol for 10 min. The hydrogel samples (1 cm × 1 cm) were sterilized under UV light for 25 min and placed in inoculated test tubes. Finally, the test tubes were incubated at 37 °C for 24 h and measured using the spectrometer UV–Vis at a wavelength of 600 nm.

To determine the counting of colony-forming units, the Muller–Hinton solution was prepared and sterilized at 120 °C. Test tubes with 4.95 mL of culture broth were inoculated with *Streptococcus mutans*, *Enterococcus faecalis*, *Pseudomonas aeruginosa*, and *Staphylococcus aureus*, respectively. The bacterial concentration was determined using the UV–vis spectrophotometer at 600 nm, and the inoculum was standardized to an optical density of 0.1 absorbance units. Hydrogel samples of 1 cm × 1 cm were cut and sterilized under UV light for 25 min. The samples were placed into the tested tubes and incubated at 37 °C for 24 h. Tubes with broth alone and hydrogel 100% cellulose were used as controls. Afterward, serial dilutions of 1:1,000,000 were made. From this dilution, 100 µL was placed in a Muller–Hinton culture medium agar dish. Streaking was then performed using a Drigaslhy loop (Chihuahua, Mexico), and the experiment was incubated at 37 °C for 24 h. Finally, the colony count number was registered.

### 4.5. Protein Adsorption

The enriched hydrogels were analyzed using the bicinchoninic acid assay to assess the protein adsorption [46]. The hydrogel samples (5 mm × 5 mm) were immersed in 1 mL of PBS containing 1 mg/mL of BSA and incubated for 4 h at 37 °C. FBS was also used. The hydrogel samples were placed in tubes with 1 mL of Dulbecco’s modified Eagle’s medium (DMEM) enriched with 10 vol% of FBS and incubated for 4 h at 37 °C. Then, the samples were rinsed 3 times for 10 min in PBS to remove the excess proteins. Finally, 2 mL of the 2 wt% solution of sodium dodecyl sulfate was added to each sample and placed in a shaking bath at 25 °C. The amount of adsorbed protein was measured at 562 nm using a calibration curve obtained from the pure protein samples [23]. Each experiment was run in triplicate, and data from the six experiments were analyzed and averaged.

### 4.6. Cell Culture

For the biocompatibility analysis, fibroblasts of mouse embryonic cells (NIH3T3) were used, as in our previous report [46]. Before the experiments, hydrogel films were cut into circles (30 mm in diameter) and sterilized with ethanol 70 vol% for 30 min. To evaluate cell adhesion and the morphology of adherent cells, a cell dispersion was prepared in the DMEM medium with a cell density of 8 × 10^3^ cm^−2^. Then, 2 mL of the cell dispersion was added to each sample and incubated at 37 °C in a CO_2_ atmosphere. The samples were analyzed after 4, 24, 48, and 72 h of cell culture to count the cell adherent numbers and calculate the cell morphology aspects. Four samples were evaluated for each hydrogel formulation, including the controls at different cell culture times. The experiments were assessed statically by one-way analysis of variance (ANOVA) and were followed by a Student’s *t*-test with a significance of *p* < 0.05.

### 4.7. Cell Morphology

The morphological parameters of the adherent cells on the hydrogel surface were evaluated using an inverted microscope (CKX4, Olympus, Tokyo, Japan). The hydrogel samples from different cell culture times were analyzed (4, 24, 48, and 72 h) [46]. After the cell culture time, the PS dish (commercially used for cell culture assays) containing the tested hydrogel sample was washed twice with 2 mL of PBS. Then, 2 mL of formaldehyde 3.7 vol% was used to fix the adherent cells to the hydrogel surface. The PS dish area was divided into four parts; each part was analyzed separately on the microscope. Twenty images were taken from each section and analyzed using the Cellsens software. Morphological parameters such as the aspect ratio, cell area, and long axis were determined. The results from this approach were based on six independent experiments.

### 4.8. Statistical Analysis

Data were analyzed using the Kruskal–Wallis statistical test and a Dunn’s multivariate comparison was carried out to determine the effectiveness of the GSE content in the hydrogel formulation. A significance of <0.05 was determined using the IBM SPSS program, version 25. Statistical analysis enabled the comparison of the mechanical properties, biocompatibility, cytotoxicity, and antimicrobial effects of the hydrogels with an increasing GSE content. All assays were conducted in triplicate, and the results included the mean values and standard deviation.

## Figures and Tables

**Table 1:** Properties of hydrogel films with variations in GSE content. Cellulose/GSE ratios (wt%/wt%) 100/0, 90/10, 70/30, and 50/50. Data are presented in mean and ± standard deviation. Samples were tested at 25 °C to obtain a reliable value.

Sample	EWC (%)	Elongation (mm)	Tensile Strength (N/mm^2^)	Shear Viscosity (CP)		Contact Angle
				6 rpm	60 rpm	
100/0	31 ± 0.882	12.67 ± 0.333	52.33 ± 0.333	410.67 ± 0.667	406.67 ± 0.882	38.33 ± 0.577
90/10	32 ± 0.577	10.33 ± 0.333	50.67 ± 0.667	402.67 ± 0.333	400.67 ± 0.333	37.33 ± 0.577
70/30	35 ± 0.882	8.33 ± 0.333	44.67 ± 0.333	386.67 ± 0.333	387.67 ± 0.333	36 ± 0.000
50/50	37 ± 0.577	6.33 ± 0.333	40 ± 0.557	363.67 ± 0.882	362.67 ± 1.202	36.33 ± 0.577

**Table 2:** Colony-forming units in dilution of 1:1,000,000 (mean ± deviation standard).

Sample	*S. mutans* Colony Number	*E. faecalis* Colony Number	*S. aureus* Colony Number	*P. aeruginosa* Colony Number
Bacteria	927.67 ± 82.276	490.00 ± 86.712	156.33 ± 20.502	263.00 ± 42.426
100/0	945.33 ± 14.189	709.00 ± 11.533	290.67 ± 88.822	248.00 ± 1.414
90/10	90.00 ± 16.823	108.00 ± 9.644	14.00 ± 2.646	261.50 ± 47.376
70/30	35.67 ± 4.33	1.67 ± 0.287	1.00 ± 0.2770	2.00 ± 0.282
50/50	0.0 ± 0.000	1.67 ± 0.155	3.00 ± 0.732	0.00 ± 0.000

## Data Availability

All data obtained during this study can be found in the research archives of the Master’s Program in Dental Sciences of the Autonomous University of Ciudad Juarez and can be requested from the corresponding author.
